# Parent’s acceptance of advanced behavior management techniques on children during dental treatment

**DOI:** 10.1186/s12887-024-05234-8

**Published:** 2024-11-25

**Authors:** Claudia Salerno, Silvia Cirio, Cinzia Maspero, Margerita Roner, Valeria D’Avola, Maria Grazia Cagetti

**Affiliations:** 1 Department of Restorative, Preventive and Pediatric Dentistry, University of Bern, Freiburgstrasse 7, 3012 Bern, Switzerland; 2 Department of Biomedical, Surgical and Dental Sciences, University of Milan, Via Beldiletto 1, 20142 Milan, Italy; 3 Graduate School for Health Sciences, University of Bern, Bern, Switzerland; 4 Fondazione IRCCS Cà Granda, Ospedale Maggiore Policlinico, University of Milan, Milan, 20100 Italy

**Keywords:** Autism spectrum disorder, Advanced behavior management techniques, Dental treatment

## Abstract

**Aim:**

This study explores the acceptance of Advanced Behavior Management Techniques (ABMTs) by parents during their children’s dental treatments, comparing the opinion of parents of neurotypical children with that of parents of children with autism spectrum disorders (ASDs).

**Methods:**

An observational cross-sectional study was conducted involving 440 parents, divided into two groups: 236 parents of neurotypical children and 204 parents of children with ASDs, recruited from pediatricians’ centers and centers for ASDs children in Northern and Southern Italy. A survey assessed their familiarity and acceptance of ABMTs, including protective stabilization, conscious sedation, and deep sedation/general anesthesia. Discrete variables were expressed as absolute and relative frequencies (%) and compared with Pearson’s chi-squared or Fisher’s exact test. Continue variables were expressed as mean ± SD and compared with the one-way ANOVA test. Heatmap and PCA analysis were used to determine possible correlations between items.

**Results:**

Parents of children with ASDs showed a higher acceptance rate of ABMTs compared to parents of neurotypical children. Overall, only 30.68% of parents knew ABMTs before the survey. Differences between the two groups of parents in acceptance of Active Stabilization in emergency settings, Passive Stabilization in routine settings, and Deep sedation/general anesthesia in both settings were observed (*p* < 0.01). Only 6.82% of parents ever used at least one ABMT on their children. Heatmap analysis revealed that parents who have accepted one of the ABMTs tend to accept the others as well.

**Conclusion:**

Differences in parental acceptance of different ABMTs was noted among the two groups of parents, with greater acceptance of ABMTs observed in the group of parents of children with ASDs. Parents of both groups have significant gaps in their knowledge of ABMTs. Therefore, increased awareness and personalized communication strategies are needed to increase acceptance of the studied techniques and, thus, facilitate access to dental care for uncooperative pediatric patients. Patient-centered behavior management strategies that meet children’s needs and parents’ preferences can contribute to the achievement of good oral health.

**Supplementary Information:**

The online version contains supplementary material available at 10.1186/s12887-024-05234-8.

## Introduction

Dental fear and dental anxiety (DFA) refer to the strong negative feelings associated with dental treatment [1]. Dental anxiety and phobia are common and affect between 3 and 10% of the population, with an increase from 6 to 21% in children and adolescents [2, 3]. Children with dental phobia or generalized anxiety often demonstrate poor cooperation during dental treatments, posing a significant challenge to ensure adequate treatment [4]. Moreover, children with special needs who do not offer adequate dental treatment cooperation further amplify pediatric dentists’ challenges [5]. Children with special needs, such as those with autism spectrum disorders (ASD), may require customized, patient-centered dental care strategies [6, 7].

Behavior management techniques (BMTs) are crucial to treating uncooperative children and can be classified into basic and advanced techniques. The effectiveness of basic techniques has been widely described in the literature, especially for techniques such as tell-show-do, positive reinforcement, vocal control, distraction, parent presence/absence, positive pre-visit imagery and PECS [8]. However, in some cases, such as those described above, the effectiveness of basic techniques alone may not be sufficient [9]. Advanced techniques (ABMTs), including protective stabilization, conscious sedation, and deep sedation/general anesthesia, are implemented to efficiently carry out dental treatments in uncooperative children and promote a positive patient attitude [9].

Protective stabilization involves immobilizing or restricting the patient’s movements to ensure safety during treatment. It can be active, involving qualified personnel or parents, or passive, using stabilization devices [10]. This procedure is particularly effective in young or uncooperative children, allowing the completion of short-duration treatments [11, 12].

Conscious sedation mainly consists of administering a mixture of gases (nitrous oxide and oxygen) through a nasal mask or drugs (e.g., benzodiazepines), which reduce anxiety while keeping the patient conscious. Sedation with nitrous oxide requires good cooperation for both the positioning of the mask and the necessary open-mouth nasal breathing. Inhalation sedation can be combined with the administration of benzodiazepines and/or protective stabilization [13].

Deep sedation and general anesthesia, on the other hand, do not require patient cooperation, inducing a state of partial or total unconsciousness, respectively. While conscious sedation can be performed by a specially trained dentist, deep sedation and general anesthesia must be administered exclusively by a specialized anesthetist, requiring, in the majority of cases, hospitalization with pre-operative exams [14]. In the case of deep sedation and general anesthesia, practitioners often opt for faster and more successful treatments, such as extractions instead of endodontic treatments, due to operational time constraints and the difficulty of scheduling closer sessions [15, 16].

Parents tend to be less accepting of ABMTs than BMTs [17]. The evidence is limited, and parents, particularly those of uncooperating children, may have divergent perspectives on the indication, possible risks, and appropriateness of advanced treatments such as protective stabilization [18–21]. An important goal in providing appropriate dental care is to establish a two-way dialogue with the child’s parents regarding the potential risks and benefits of ABMTs that can be employed [22]. Parents’ opinions should be taken into account by dentists, while respecting guideline recommendations, in order to choose the most appropriate ABMTs to be used in each individual patient and situation [23].

Given the importance of parents’ thoughts on behavior management usage in the treatment plan, investigating their point of view is vital when determining behavior guidance application priorities. Their willingness to accept children’s behavior management strategies may be determined by how they are informed and their personal experience with these methods. Based on these premises, the present study aims to investigate the degree of acceptance of advanced behavior management techniques by parents of children with autism spectrum disorders and to compare it with that of parents of neurotypical children.

## Materials and methods

The study was designed as an observational, questionnaire-based, cross-sectional study; it complied with the Declaration of Helsinki and was performed according to ethics committee approval (Ethics Committee Board of the University of Milan, Milan, Italy, N° N. 0024829 on 01/06/2021).

The questionnaire was developed based on two previously validated questionnaires [18, 24] and consisted of 19 items:

- 3 items on demographic characteristics, including sex, educational level, and origin of the parent who was filling out the questionnaire;
- 4 items on characteristics of the child, including age, where he/she receives dental care, level of cooperation and if he/she is affected by ASDs;
- 12 items on parent’s familiarity with ABMTs, opinion on each type of ABMTs (active and passive protective stabilization, conscious sedation, deep sedation/general anesthesia) during routine or emergency dental care, and previous experience about ABMTs.

The English version of the questionnaire can be found in Supplementary file S2. It was translated from English into Italian by two native Italian-speaking translators who were fluent in English and experienced in the topic. After the translation, a consensus version was identified; then, it was retro-translated into English by a third person, a native English-speaking translator not involved in the study, to ensure the accuracy and comparability of the translation. A quantitative analysis of the accuracy of the questionnaire was performed by submitting it to 12 experts in pediatric dentistry (4 self-employed dentists specialized in Pediatric Dentistry with more than 5 years of working experience, 4 academics, and 4 clinical researchers). The quantitative content validity of each item was assessed using the Content Validity Index (CVI) and the Content Validity Ratio (CVR) [25]. Based on the experts’ opinions, the CVI and S-CVR were found to be 1.00 (Supplementary file S1).

The final Italian version was pre-tested in January 2020 for comprehensibility on a small sample of 20 parents (10 males and 10 females) not included in the survey. After completing the questionnaire, they were contacted to find out if they had experienced any difficulty in understanding the questions and were given a comprehension score from 1 (extreme difficulty) to 5 (no difficulty). A result of 4.91 ± 0.06 was obtained.

An online version of the anonymous questionnaire was created using Google Form (Google LLC, Mountain View, CA, USA) and made accessible via a QR code. A convenience sample of pediatricians’ centers and centers for ASDs children in Northern Italy (Milan) and Southern Italy (Naples) was selected by an online search and contacted by phone. In those available, the flyer with the QR code was distributed. Before the first question, a description of the purpose of the study and of the ABMTs was included and parents were asked to sign an informed consent to participate in the study and, in accordance with Italian law, a second informed consent concerning the processing of personal data. If they did not sign both consents, the questionnaire was automatically closed.

The following inclusion criteria were used for enrolment:

- signing the online informed consent;
- be a parent of at least one child aged between 1 and 14 years;
- be resident in Italy.

All subjects who did not meet the above inclusion criteria were excluded from the survey. In addition, parents with children with disabilities other than ASDs were excluded.

The sample size was calculated based on data in the literature. Considering that it’s reported a difference of 21% in parents’ opinions on protective stabilizations (47% among parents of children with ASDs and 26% among parents of neurotypical peers), a sample size of 134 parents for each group was calculated, with two-tail test, with a power of 95% and significance level of 0.05 [17].

The survey was conducted from January 2022 until December 2022. Data were collected in January 2023.

### Statistical analysis

The deidentified data were downloaded from the survey site, imported into a Microsoft Excel spreadsheet, and quality-checked to ensure accuracy. Data from participants whose questionnaires were incomplete or whose responses to sentinel questions were inconsistent were excluded. Descriptive statistics were calculated for all items to provide an overview of the results. Analyses were conducted using STATA SE 18.0 for MAC. The demographic item "Is your child affected by ASD" was used to divide the sample into two groups: questionnaires filled out by parents of children with ASDs and those by parents of neurotypical peers. Discrete variables were expressed as absolute and relative frequencies (%) and compared with Pearson’s chi-squared or Fisher’s exact test. Continue variables were expressed as mean ± SD and compared with one-way ANOVA test. The alpha risk was set to 5%, and two-tailed tests were used.

The relationship between the questionnaire items was calculated using Pearson’s correlation coefficients. Heatmap analysis was used to visualize the correlations between the categorical variables, after recoding them numerically (see Supplementary file S3). Principal component analysis (PCA) was conducted to identify patterns among the survey variables (PCA) [26]. The data were checked for multicollinearity using the Belsley–Kuh–Welsch technique. The heteroskedasticity and normality of the residuals were assessed using the White test and the Shapiro–Wilk test, respectively. The interaction model (likelihood ratio test statistic) evaluated potential effects modifiers.

## Results

The questionnaire was opened by 476 subjects, 23 parents did not sign the informed consent (4.83%), 7 (1.47%) did not complete the questionnaire, and 6 (1.26%) did not answer the questionnaire properly (inconsistent sentinel questions). Therefore, questionnaires filled out by 440 parents were included and analyzed. Due to the study design and questionnaire distribution, no response rate was evaluated. The study sample included 236 parents of neurotypical children (53.64%) and 204 parents of children with ASDs (53.64%). The mean age of children was 7.61 _95_CI [7.34; 7.88], with no difference in the two groups (*p* = 0.58). Most responders were mothers (77.59%) born in Europe (88.18%). Different distribution of parents’ birthplace was observed in the two groups (*p* < 0.01). Almost half of the sample of parents (41.36%) have brought their child to a private dentist, with significant differences in the two groups (60.17% vs. 19.61%), while 32.05% have never brought their children to the dentist (22.46% vs. 43.14%). No differences were found between the two groups regarding parents’ educational level (*p* = 0.73) (Table 1). Parents of children with ASDs judged worse their children’s cooperation during dental treatment (1.71 ± 1.75 *vs* 3.3 ± 1.92; *p* < 0.01).

**Table 1:** Demographic characteristics of the responders

	**Total**	**Neurotypical peers**	**Children with ASD**	***P* Value**
	*N* = 440	*N* = 236	*N* = 204	
	N (%)	N (%)	N (%)	
**Who is filling out this questionnaire?**				
Father	97 (22.05)	47 (19.92)	50 (24.51)	0.30
Mother	343 (77.95)	189 (80.08)	154 (75.49)	
**Which region are you from?**				
Europe	388 (88.18)	226 (95.76)	162 (79.41)	< 0.01
South America	38 (8.64)	5 (2.12)	33 (16.18)	
Africa	10 (2.27)	5 (2.12)	5 (2.45)	
Asia	4 (0.91)	0 (0.00)	4 (1.96)	
**What is your level of education?**				
Primary school degree	4 (0.91)	2 (0.85)	2 (0.98)	0.73
Middle school degree	58 (13.18)	29 (12.29)	29 (14.22)	
High school degree	205 (46.59)	105 (44.49)	100 (49.02)	
Bachelor’s degree	124 (28.18)	72 (30.51)	52 (25.49)	
Post-bachelor’s degree	49 (11.14)	28 (11.86)	21 (10.29)	
**Where did your child receive dental care?**				
Public dental clinic (hospital or clinic affiliated with SSR)	102 (23.18)	37 (15.68)	72 (35.29)	< 0.01
Private dental clinic	182 (41.36)	146 (61.86)	44 (21.57)	
He/She’s never been to the dentist	141 (32.05)	53 (22.46)	88 (43.14)	
**How do you think is his/her cooperation during dental care?**				
Totally uncooperative	19 (4.32)	3 (1.27)	16 (7.84)	< 0.01
Poorly cooperative	30 (6.82)	4 (1.69)	26 (12.75)	
A little cooperative	41 (9.32)	15 (6.36)	26 (12.75)	
Quite cooperative	120 (27.27)	83 (35.17)	37 (18.14)	
Very cooperative	89 (20.23)	78 (33.05)	11 (5.39)	
**How old is your child?**				
**Mean (SD)**	7.61 (2.90)	7.44 (3.02)	7.80 (2.76)	0.58
**Median**	8.00	8.00	7.80 (2.76)	
**Min—Max**	1.00—14.00	1.00; 14.00	1.00; 14.00	
**95% confidence interval**	7.33 -7.88	7.06–7.82	7.42–8.18	

*N* number, *SD* standard deviation

Only 30.68% of parents knew ABMTs before the survey and their acceptance is described in Table 2. Passive stabilization was judged acceptable/extremely acceptable by 45.00% and 67.73% of parents in routine and emergency settings, respectively. Deep sedation and general anesthesia were judged acceptable/extremely acceptable by 46.59% and 85.46% of parents in routine and emergency settings, respectively. Differences in parental acceptance of Active Stabilization in emergency settings, Passive Stabilization in routine settings and Deep sedation/general anesthesia in both settings were observed (*p* < 0.01).

**Table 2:** Parental knowledge and acceptance of ABMTs

	**Total**	**Neurotypical children**	**Children with ASD**	***P* Value**
	*N* = 440	*N* = 236	*N* = 204	
	N (%)	N (%)	N (%)	
**Were you already familiar with advanced behavior management techniques before today?**				
Yes	135 (30.68)	71 (30.08)	64 (31.37)	0.85
No	305 (69.32)	165 (69.92)	140 (68.63)	
**What do you think about active protective stabilization during routine dental care?**				
Totally unacceptable	14 (3.18)	7 (2.97)	7 (3.43)	0.08
Unacceptable	34 (7.73)	22 (9.32)	12 (5.88)	
Indifferent	48 (10.91)	32 (13.56)	16 (7.84)	
Acceptable	239 (54.32)	115 (48.73)	124 (60.78)	
Totally acceptable	105 (23.86)	60 (25.42)	45 (22.06)	
**Mean (SD)**	3.88 (0.96)	3.84 (1.00)	3.92 (0.92)	0.48
**Median**	4.00	4.00	4.00	
**Min—Max**	1.00—5.00	1.00—5.00	1.00—5.00	
**95% Confidence Interval**	3.79 -3.97	3.71–3.97	3.79–4.05	
**What do you think about active protective stabilization during emergency dental care?**				
Totally unacceptable	11 (2.50)	2 (0.85)	9 (4.41)	0.01
Unacceptable	35 (7.95)	13 (5.51)	22 (10.78)	
Indifferent	28 (6.36)	19 (8.05)	9 (4.41)	
Acceptable	226 (51.36)	120 (50.85)	106 (51.96)	
Totally acceptable	140 (31.82)	82 (34.75)	58 (28.43)	
**Mean (SD)**	4.02 (0.96)	4.13 (0.84)	3.89 (1.07)	0.05
**Median**	4.00	4.00	4.00	
**Min—Max**	1.00—5.00	1.00—5.00	1.00—5.00	
**95% Confidence Interval**	3.93 -4.11	4.02–4.24	3.74–4.04	
**How do you judge passive protective stabilization during routine dental care?**				
Totally unacceptable	55 (12.50)	32 (13.56)	23 (11.27)	< 0.01
Unacceptable	126 (28.64)	76 (32.2)	50 (24.51)	
Indifferent	61 (13.86)	41 (17.37)	20 (9.8)	
Acceptable	160 (36.36)	67 (28.39)	93 (45.59)	
Totally acceptable	38 (8.64)	20 (8.47)	18 (8.82)	
**Mean (SD)**	3.00 (1.22)	2.86 (1.21)	3.16 (1.22)	< 0.01
**Median**	3.00	3.00	4.00	
**Min—Max**	1.00—5.00	1.00—5.00	1.00—5.00	
**95% Confidence Interval**	2.89–3.11	2.71–3.01	2.99–3.33	
**How do you judge passive protective stabilization during emergency dental care?**				
Totally unacceptable	34 (7.73)	15 (6.36)	19 (9.31)	0.13
Unacceptable	59 (13.41)	34 (14.41)	25 (12.25)	
Indifferent	49 (11.14)	34 (14.41)	15 (7.35)	
Acceptable	217 (49.32)	112 (47.46)	105 (51.47)	
Totally acceptable	81 (18.41)	41 (17.37)	40 (19.61)	
**Mean (SD)**	3.57 (1.16)	3.55 (1.12)	3.60 (1.20)	0.39
**Median**	4.00	4.00	4.00	
**Min—Max**	1.00—5.00	1.00—5.00	1.00—5.00	
**95% Confidence Interval**	3.46 -3.68	3.41–3.69	3.43–3.77	
**How do you judge conscious sedation during routine dental care?**				
Totally unacceptable	12 (2.73)	8 (3.39)	4 (1.96)	0.12
Unacceptable	34 (7.73)	19 (8.05)	15 (7.35)	
Indifferent	64 (14.55)	41 (17.37)	23 (11.27)	
Acceptable	254 (57.73)	123 (52.12)	131 (64.22)	
Totally acceptable	76 (17.27)	45 (19.07)	31 (15.20)	
**Mean (SD)**	3.79 (0.91)	3.75 (0.97)	3.83 (0.84)	0.46
**Median**	4.00	4.00	4.00	
**Min—Max**	1.00—5.00	1.00—5.00	1.00—5.00	
**95% confidence interval**	3.71 -3.88	3.63–3.87	3.71–3.95	
**How do you judge conscious sedation during emergency dental care?**				
Totally unacceptable	5 (1.14)	2 (0.85)	3 (1.47)	0.27
Unacceptable	16 (3.64)	6 (2.54)	10 (4.90)	
Indifferent	29 (6.59)	18 (7.63)	11 (5.39)	
Acceptable	240 (54.55)	122 (51.69)	118 (57.84)	
Totally acceptable	150 (34.09)	88 (37.29)	62 (30.39)	
**Mean (SD)**	4.17 (0.79)	4.22 (0.76)	4.11 (0.82)	0.16
**Median**	4.00	4.00	4.00	
**Min—Max**	1.00—5.00	1.00—5.00	1.00—5.00	
**95% Confidence Interval**	4.09 -4.24	4.12–4.32	4.00–4.22	
**How do you judge deep sedation or general anesthesia during routine dental care?**				
Totally unacceptable	53 (12.05)	35 (14.83)	18 (8.82)	< 0.01
Unacceptable	127 (28.86)	82 (34.75)	45 (22.06)	
Indifferent	55 (12.50)	32 (13.56)	23 (11.27)	
Acceptable	161 (36.59)	66 (27.97)	95 (46.57)	
Totally acceptable	44 (10.00)	21 (8.90)	23 (11.27)	
**Mean (SD)**	3.04 (1.24)	2.81 (1.24)	3.29 (1.19)	< 0.01
**Median**	3.00	3.00	4.00	
**Min—Max**	1.00—5.00	1.00—5.00	1.00—5.00	
**95% confidence interval**	2.92 -3.15	2.65–2.97	3.13–3.45	
**How do you judge deep sedation or general anesthesia during emergency dental care?**				
Totally unacceptable	7 (1.59)	7 (2.97)	0 (0.00)	< 0.01
Unacceptable	26 (5.91)	20 (8.47)	6 (2.94)	
Indifferent	31 (7.05)	19 (8.05)	12 (5.88)	
Acceptable	261 (59.32)	126 (53.39)	135 (66.18)	
Totally acceptable	115 (26.14)	64 (27.12)	51 (25.00)	
**Mean (SD)**	4.03 (0.84)	3.93 (0.98)	4.13 (0.64)	0.17
**Median**	4.00	4.00	4.00	
**Min—Max**	1.00—5.00	1.00—5.00	1.00—5.00	
**95% Confidence Interval**	3.95 -4.10	3.80–4.06	4.04–4.22	
**Have you ever allowed advanced behaviour management techniques for dental treatment to be performed on your child?**				
Yes	30 (6.82)	7 (2.97)	23 (11.27)	< 0.01
No	410 (93.18)	229 (97.03)	181 (88.73)	
**If yes, which one?**				
Active protective stabilization	15 (3.41)	4 (1.69)	11 (5.39)	0.04
Passive protective stabilization	10 (2.27)	1 (0.42)	9 (4.41)	0.01
Conscious sedation	11 (2.50)	2 (0.85)	9 (4.41)	0.03
Deep sedation or general anesthesia	8 (1.82)	1 (0.42)	7 (3.43)	0.01
**Now that you know the different advanced behaviour management techniques, would you have preferred another one to be used?**	*N* = 30	*N* = 7	*N* = 23	
Yes	11 (36.67)	3 (42.86)	8 (34.78)	> 0.99
No	19 (63.33)	4 (57.14)	15 (65.22)	

*N* number, *SD* standard deviation

Only 6.82% of parents ever used at least one ABMT on their children, half of them used active stabilization, and 36.67% of them, after knowing all the techniques, would have preferred to use another one.

**Fig. 1:**
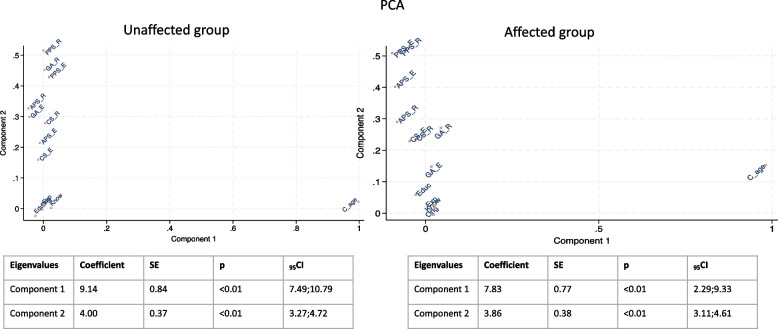
Principal component analysis in the two groups. Legend: BMT= Behavioral Management Technique; Edu=educational level; Orig=birthplace; Know=BMT knowledge; Exp= BMT experience; APS_R=Active Protective Stabilization during Routine dental care; APS_E=Active Protective Stabilization during Emergency dental care; PPS_R=Passive Protective Stabilization during Routine dental care; PPS_E=Passive Protective Stabilization during Emergency dental care; CS_R=Conscious Sedation during Routine dental care; CS_P=Passive Conscious Sedation during Emergency dental care; GA_R= Deep sedation/General Anesthesia during Routine dental care; GA_E= Deep sedation/General Anesthesia during Emergency dental care

**Fig. 2:**
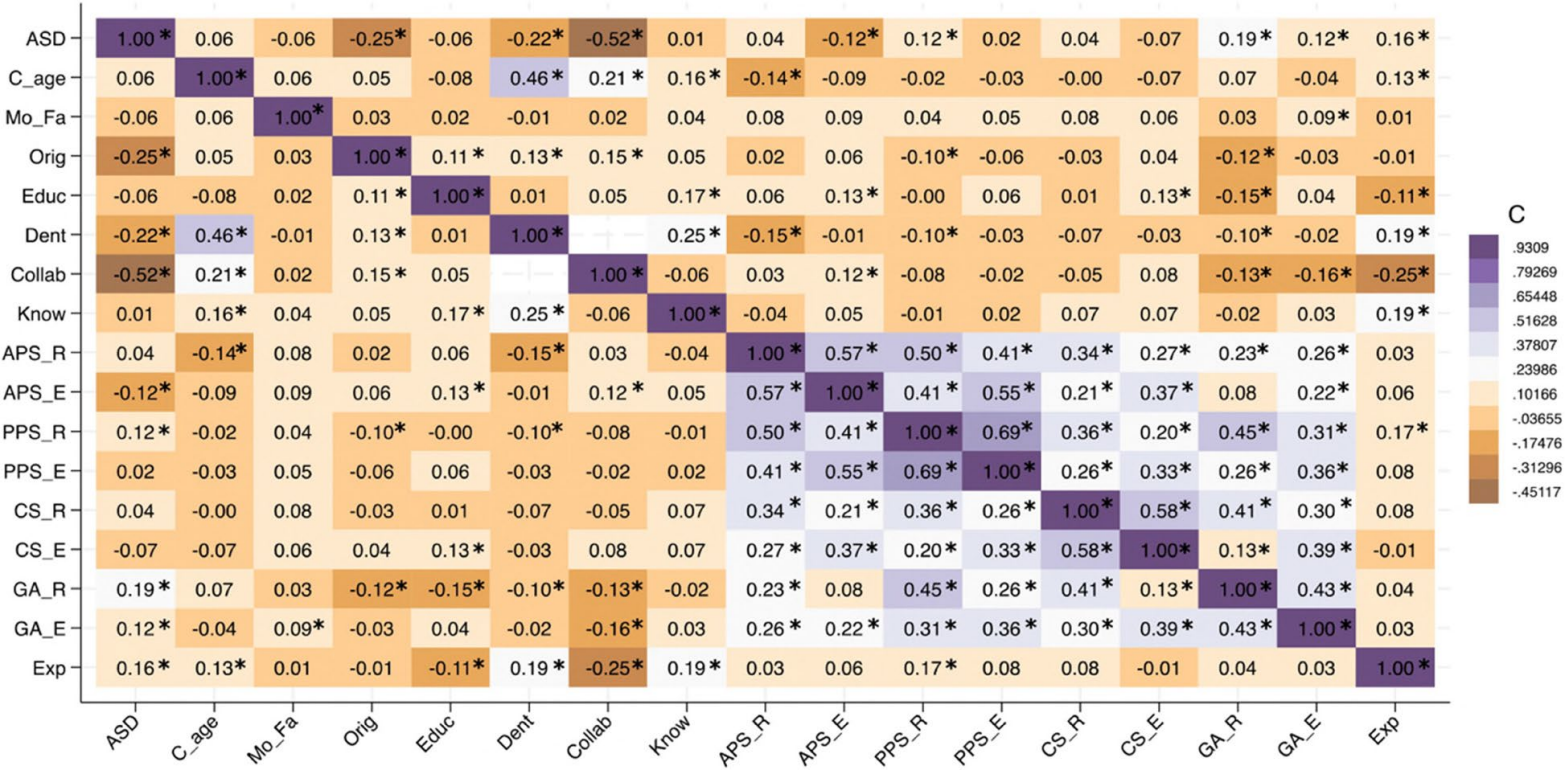
Correlation matrix. Legend: The colored squares show the correlation coefficient between two variables, with deeper color representing a stronger correlation. Blue shades denote positive correlations, while brown shades show negative correlations. Parents who have accepted one of the ABMTs tend to accept the others as well. BMT= Behavioral Management Technique; ASD= Autism Spectrum Disorder; C_age= children’s age; Mo_Fa= parent’s sex; Orig= birth place; Edu= educational level; Know= BMT knowledge; Dent=children cared by a dentist; Collab= childrens’ collaboration degree; APS_R= Active Protective Stabilization during Routine dental care; APS_E= Active Protective Stabilization during Emergency dental care; PPS_R= Passive Protective Stabilization during Routine dental care; PPS_E= Passive Protective Stabilization during Emergency dental care; CS_R= Conscious Sedation during Routine dental care; CS_P= Passive Conscious Sedation during Emergency dental care; GA_R= Deep sedation/ General Anesthesia during Routine dental care; GA_E= Deep sedation/ General Anesthesia during Emergency dental care; Exp= BMT experience; *= *p*<0.01

PCA was performed on the data set in the two groups: the parents of neurotypical children and of those with ASD (Fig. 1). In the two PCAs, the first two eigenvalues, obtained from the distance matrix between subgroups, collectively accounted for more than 66.0% of the total variance (70.54%; 49.06 and 21.48%, respectively in the group of parents with neurotypical children, and 66.68% in the group with children with ASDs 45.95% and 22.73% two eigenvalues respectively). Figure 2 displays the Orthogonal Rotation (Varimax) of the two groups’ first principal components. In the parents’ sample of unaffected children, the place of birth, level of education, previous knowledge, and experience of ABMTs tended to form a separate cluster; also, Deep sedation/General Anesthesia in routine settings and Passive Stabilization in both routine and emergency settings acceptance tended to create another cluster; however, children’s age was separated from the other variables. Similar results were observed in parents’ sample of children with ASD: the place of birth, the level of education, previous knowledge, and experience of ABMTs tended to form a separate cluster. Also, Passive stabilization in both settings tended to create another cluster, and in this group, children’s age was separated from the other variables. PCA was also performed in the total sample; however, the first two eigenvalues accounted for less than 66.0% of the total variance.

The correlation matrix shown in Fig. 2 visually depicts the connections between the questionnaire’s items. Using the matrix makes it possible to detect correlation patterns between these variables.

## Discussion

This study investigated the degree of acceptance by parents of children with or without ASDs of advanced BMTs, which can be used to carry out dental treatments in uncooperative children. The survey has involved 440 parents with equal representation from parents of neurotypical children and those with ASD. A significant portion of parents of children with ASDs opted for the public dental facility for their children, unlike parents of neurotypical children who opted for a private dentist. Additionally, a significant number of children with ASDs has never been visited by the dentist, indicating potential gaps in access to dental care. Parents of children with ASDs reported poorer cooperation during dental treatment compared to parents of neurotypical peers, highlighting the challenges faced in managing dental anxiety and behavior in this population [27]. Moreover, only a minority of all parents knew ABMTs before the survey, indicating the need to increase awareness about the available techniques. Differences in parental acceptance of different ABMTs was noted among the two groups of parents. Passive stabilization was considered more acceptable in both routine and emergency settings by parents of children with ASDs compared to those of neurotypical peers. The percentage of parents in both groups willing to accept active and passive restraint both in routine and emergency setting was higher than that described in a similar study conducted in Germany [18]. Moreover, conscious sedation and deep sedation/general anesthesia in routine settings were judged more acceptable by parents of children with ASD. A small percentage of parents were allowed to use ABMTs on their children, with a clear prevalence among children with ASDs for all ABMTs.

In both groups, factors such as place of birth, level of education, previous knowledge, and experience of ABMTs tended to cluster together. Interestingly, children’s age appeared clearly separated from the other variables, suggesting a potential lack of influence on parental acceptance of ABMTs. This finding is surprising, as younger children are typically less cooperative and more anxious in the dental environment [28, 29]. However, this result agrees with previous literature [20, 30]. In parents of children with ASDs, it could be hypothesized that the specific challenges associated with the spectrum may overshadow the impact of age, making it a less significant factor in parental decision-making. Parents might probably base their acceptance of ABMTs more on their child’s overall behavioral tendencies than their age, reflecting a more individualized approach to decision-making. Furthermore, the correlation matrix showed that parents who accepted one ABMT tended to accept the others, suggesting consistency in their attitudes toward the different techniques.

The preference of parents of children with ASDs for public dental facilities over private dentists reflects the need to find affordable solutions, considering the challenges that families with children with ASDs may face [31]. Although the supply of public dentistry is very limited in Italy, and most citizens of all ages turn to private dentists for treatment, these latter are generally neither trained nor organized to treat children with special needs who require a well-trained team to manage the specific behaviors that the spectrum entails. The fact that more than a third of the sample of children with ASDs have never been to a dentist is in line with previous research, highlighting inequalities in access to health services [32]. Families face significant obstacles in obtaining appropriate dental care for their children with ASD, not only because of the scarcity of specialized centers but also because of limited knowledge of advanced behavior management techniques that can facilitate care [33]. In a context where many families are unaware of the options available to manage anxiety and behavior during dental care, it is essential to inform and support families to make their approach to the dental environment easier.

Differences in the acceptance of ABMTs between parents of children with ASDs and their neurotypical peers emphasize the importance of understanding individual preferences and needs when implementing behavior management strategies. These findings can inform clinical practices and guide the development of personalized protocols that consider patients’ preferences and needs [17]. Moreover, they can be used to inform the development of targeted interventions that specifically address the determinants of parents’ decision-making about dental care for their children, thereby improving access and quality of care. In addition, the consistency observed in parental attitudes towards different ABMTs suggests a coherent framework in parental decision-making. Communication strategies and clinical approaches that take into account shared preferences and trends among parents may facilitate the planning and implementation of dental care for uncooperative children, both with and without ASDs [20].

The present findings should be interpreted cautiously due to limitations, particularly sampling and response bias. The sample was recruited in two geographically restricted areas of Italy using a recruitment method that reached only parents attending a limited number of pediatric outpatient clinics and autism centers without randomization of the sample. Although efforts were made to ensure a fair representation of parents of neurotypical children and those with ASD, the sample may not be fully representative of a broader population of parents. Furthermore, response bias may have influenced the results, as parents who chose to participate in the survey may have stronger opinions on ABMTs or more experience with dental care than those who did not, thus influencing the results. Reliance on self-reported data is another possible limitation, as it may introduce recall or social desirability biases [34, 35].

Although protective stabilization is an effective technique to carry out noninvasive and minimally invasive treatments in uncooperative children, its ethical implications continue to be scrutinized. In the present survey, protective stabilization resulted in a less acceptable technique, especially among parents of neurotypical children. On the one hand, protective stabilization helps minimize the risk of injury to the child and dental staff, ensuring that necessary dental procedures can be completed effectively [36]. Still, on the other side, some parents may express concerns about its potential psychological impact and violation of the child’s autonomy. Parents may fear that restraining their children could exacerbate their anxiety and lead to negative associations with dental care, ultimately hindering their long-term oral health outcomes [37]. Dentists must communicate effectively with parents, explaining the rationale behind the use of protective stabilization, its potential benefits, and any available alternatives. In addition, dentists should strive to create a supportive and compassionate environment in which parents feel empowered to express their concerns and actively participate not only in the decision-making process but, if available, in the act of restraint itself to reassure the child with their presence. Moreover, restraint should occur whenever possible after the child has been prepared to familiarize them with the dental environment’s people, instruments, noises, and smells. This preparation makes children less anxious and parents more likely to accept a restraint strategy. Nevertheless, research is needed to evaluate protective stabilization’s long-term outcomes and ethical implications.

The results of this study provide dentists with helpful information to improve their clinical practice when treating uncooperative children, especially those with ASD. Improving parents’ knowledge and understanding of ABMTs enables them to choose the most accepted method under the practitioner’s guidance and thus to face more awareness of their children’s dental care. This goal can be achieved with educational materials, pre-visit consultations, and clear and effective communication. Training dental staff is also essential; to improve the quality of care and meet the needs of each child and family, the dental team should receive specific training on managing uncooperative children and children with ASDs, including in-depth knowledge of all ABMTs.

Only by addressing the concerns of parents and dental professionals and by promoting open communication and shared decision-making can the dental community work towards providing safe, effective, and patient-centered care for uncooperative children needing dental treatment.

The present survey results draw attention to a relatively under-explored area, but one crucial to improving equity, access, and quality of care. A greater understanding of the acceptance of ABMTs by parents of children with ASDs may enable dental staff to gain awareness of which techniques are more accepted and which are less. Important differences emerge between the two parent groups, particularly in accepting techniques such as passive stabilization and sedation. Furthermore, the study highlights a significant lack of knowledge among parents regarding ABMTs, particularly among those who have children with ASDs. This finding indicates the need for targeted educational efforts to improve parents’ understanding of these techniques, which could lead to better dental care experiences for uncooperative children.

## Supplementary Information

Supplementary Material 1.

## Data Availability

The datasets generated and/or analyzed during this study are not included in this published article or in a publicly available repository and will be made available on request.
