# Supplementary material for: Parent’s acceptance of advanced behavior management techniques on children during dental treatment
Source: BMC Pediatr. 2024 Nov 25;24:764. doi: 10.1186/s12887-024-05234-8 (PMC11587585; doi:10.1186/s12887-024-05234-8)
Supplement: Supplementary file 1 — Supplementary Material 1. [file 12887_2024_5234_MOESM1_ESM.docx]

Supplementary files

| File | Page |
| --- | --- |
| File S1: Content Validity Index (CVI) and the Content Validity Ratio (CVR) | 2 |
| File S2: English verision of the questionnaire | 5 |
| File S3: Variables re-coding in numerical variables | 8 |

File S1: Content Validity Index (CVI) and the Content Validity Ratio (CVR)

|  | **Question** | **CVR** | **Relevancy** | **Clarity** | **CVI** |
| --- | --- | --- | --- | --- | --- |
| **1** | **Who is filling this questionnaire?** |  |  |  |  |
|  | Mather | 1.00 | 1.00 | 1.00 | 1.00 |
|  | Father | 1.00 | 1.00 | 1.00 | 1.00 |
| **2** | **Which region are you from?** |  |  |  |  |
|  | Europe | 1.00 | 1.00 | 1.00 | 1.00 |
|  | Asia | 1.00 | 1.00 | 1.00 | 1.00 |
|  | North America | 1.00 | 1.00 | 1.00 | 1.00 |
|  | South America | 1.00 | 1.00 | 1.00 | 1.00 |
|  | Africa | 1.00 | 1.00 | 1.00 | 1.00 |
|  | Oceania | 1.00 | 1.00 | 1.00 | 1.00 |
| **3** | **What is your level of education?** |  |  |  |  |
|  | Primary school degree | 1.00 | 1.00 | 1.00 | 1.00 |
|  | Middle school degree | 1.00 | 1.00 | 1.00 | 1.00 |
|  | High school degree | 1.00 | 1.00 | 1.00 | 1.00 |
|  | Bachelor's degree | 1.00 | 1.00 | 1.00 | 1.00 |
|  | Post-bachelor’s degree | 1.00 | 1.00 | 1.00 | 1.00 |
| **4** | **How old is your child? (Open-ended question)** | 1.00 | 1.00 | 1.00 | 1.00 |
| **5** | **Does your child have a disability?** |  |  |  |  |
|  | Yes | 1.00 | 1.00 | 1.00 | 1.00 |
|  | No | 1.00 | 1.00 | 1.00 | 1.00 |
| **6** | **Where did your child receive dental care?** |  |  |  |  |
|  | Private dental clinic | 1.00 | 1.00 | 1.00 | 1.00 |
|  | Public dental clinic (hospital or clinic affiliated with SSR) | 1.00 | 1.00 | 1.00 | 1.00 |
|  | He/She 's never been to the dentist | 1.00 | 1.00 | 1.00 | 1.00 |
| **7** | **How do you think is his/her cooperation during dental care?** |  |  |  |  |
|  | Totally uncooperative | 1.00 | 1.00 | 1.00 | 1.00 |
|  | Poorly cooperative | 1.00 | 1.00 | 1.00 | 1.00 |
|  | A little cooperative | 1.00 | 1.00 | 1.00 | 1.00 |
|  | Quite cooperative | 1.00 | 1.00 | 1.00 | 1.00 |
|  | Very cooperative | 1.00 | 1.00 | 1.00 | 1.00 |
|  | He/She 's never been to the dentist | 1.00 | 1.00 | 1.00 | 1.00 |
| **8** | **Were you already familiar with advanced behavior management techniques before today?** |  |  |  |  |
|  | Yes | 1.00 | 1.00 | 1.00 | 1.00 |
|  | No | 1.00 | 1.00 | 1.00 | 1.00 |
| **9** | **How do you think about ACTIVE PROTECTIVE STABILIZATION during ROUTINE dental care?** |  |  |  |  |
|  | Totally unacceptable | 1.00 | 1.00 | 1.00 | 1.00 |
|  | Unacceptable | 1.00 | 1.00 | 1.00 | 1.00 |
|  | Indifferent | 1.00 | 1.00 | 1.00 | 1.00 |
|  | Acceptable | 1.00 | 1.00 | 1.00 | 1.00 |
|  | Totally acceptable | 1.00 | 1.00 | 1.00 | 1.00 |
| **10** | **How do you think about ACTIVE PROTECTIVE STABILIZATION during EMERGENCY dental care?** |  |  |  |  |
|  | Totally unacceptable | 1.00 | 1.00 | 1.00 | 1.00 |
|  | Unacceptable | 1.00 | 1.00 | 1.00 | 1.00 |
|  | Indifferent | 1.00 | 1.00 | 1.00 | 1.00 |
|  | Acceptable | 1.00 | 1.00 | 1.00 | 1.00 |
|  | Totally acceptable | 1.00 | 1.00 | 1.00 | 1.00 |
| **11** | **How do you think about PASSIVE PROTECTIVE STABILIZATION during ROUTINE dental care?** |  |  |  |  |
|  | Totally unacceptable | 1.00 | 1.00 | 1.00 | 1.00 |
|  | Unacceptable | 1.00 | 1.00 | 1.00 | 1.00 |
|  | Indifferent | 1.00 | 1.00 | 1.00 | 1.00 |
|  | Acceptable | 1.00 | 1.00 | 1.00 | 1.00 |
|  | Totally acceptable | 1.00 | 1.00 | 1.00 | 1.00 |
| **12** | **How do you think about PASSIVE PROTECTIVE STABILIZATION during EMERGENCY dental care?** |  |  |  |  |
|  | Totally unacceptable | 1.00 | 1.00 | 1.00 | 1.00 |
|  | Unacceptable | 1.00 | 1.00 | 1.00 | 1.00 |
|  | Indifferent | 1.00 | 1.00 | 1.00 | 1.00 |
|  | Acceptable | 1.00 | 1.00 | 1.00 | 1.00 |
|  | Totally acceptable | 1.00 | 1.00 | 1.00 | 1.00 |
| **13** | **How do you think about CONSCIOUS SEDATION during ROUTINE dental care?** |  |  |  |  |
|  | Totally unacceptable | 1.00 | 1.00 | 1.00 | 1.00 |
|  | Unacceptable | 1.00 | 1.00 | 1.00 | 1.00 |
|  | Indifferent | 1.00 | 1.00 | 1.00 | 1.00 |
|  | Acceptable | 1.00 | 1.00 | 1.00 | 1.00 |
|  | Totally acceptable | 1.00 | 1.00 | 1.00 | 1.00 |
| **14** | **How do you think about CONSCIOUS SEDATION during EMERGENCY dental care?** |  |  |  |  |
|  | Totally unacceptable | 1.00 | 1.00 | 1.00 | 1.00 |
|  | Unacceptable | 1.00 | 1.00 | 1.00 | 1.00 |
|  | Indifferent | 1.00 | 1.00 | 1.00 | 1.00 |
|  | Acceptable | 1.00 | 1.00 | 1.00 | 1.00 |
|  | Totally acceptable | 1.00 | 1.00 | 1.00 | 1.00 |
| **15** | **How do you think about DEEP SEDATION or GENERAL ANESTHESIA during ROUTINE dental care?** |  |  |  |  |
|  | Totally unacceptable | 1.00 | 1.00 | 1.00 | 1.00 |
|  | Unacceptable | 1.00 | 1.00 | 1.00 | 1.00 |
|  | Indifferent | 1.00 | 1.00 | 1.00 | 1.00 |
|  | Acceptable | 1.00 | 1.00 | 1.00 | 1.00 |
|  | Totally acceptable | 1.00 | 1.00 | 1.00 | 1.00 |
| **16** | **How do you think about DEEP SEDATION or GENERAL ANESTHESIA during EMERGENCY dental care?** |  |  |  |  |
|  | Totally unacceptable | 1.00 | 1.00 | 1.00 | 1.00 |
|  | Unacceptable | 1.00 | 1.00 | 1.00 | 1.00 |
|  | Indifferent | 1.00 | 1.00 | 1.00 | 1.00 |
|  | Acceptable | 1.00 | 1.00 | 1.00 | 1.00 |
|  | Totally acceptable | 1.00 | 1.00 | 1.00 | 1.00 |
| **17** | **Have you ever allowed advanced behaviour management techniques to be used on your child to carry out dental care?** |  |  |  |  |
|  | Yes | 1.00 | 1.00 | 1.00 | 1.00 |
|  | No | 1.00 | 1.00 | 1.00 | 1.00 |
| **18** | **If yes, which one?** |  |  |  |  |
|  | Active protective stabilization | 1.00 | 1.00 | 1.00 | 1.00 |
|  | Passive protective stabilization | 1.00 | 1.00 | 1.00 | 1.00 |
|  | Conscious sedation | 1.00 | 1.00 | 1.00 | 1.00 |
|  | Deep sedation or general anaesthesia | 1.00 | 1.00 | 1.00 | 1.00 |
| **19** | **Now that you know the different advanced behaviour management techniques, would you have preferred to use another one?** |  |  |  |  |
|  | Yes | 1.00 | 1.00 | 1.00 | 1.00 |
|  | No | 1.00 | 1.00 | 1.00 | 1.00 |
|  | **Total** | **1.00** | **1.00** | **1.00** | **1.00** |

File S2: English version of the questionnaire

|  | **Questions** |
| --- | --- |
| **1** | **Who is filling this questionnaire?** |
|  | Mather |
|  | Father |
| **2** | **Which region are you from?** |
|  | Europe |
|  | Asia |
|  | North America |
|  | South America |
|  | Africa |
|  | Oceania |
| **3** | **What is your level of education?** |
|  | Primary school degree |
|  | Middle school degree |
|  | High school degree |
|  | Bachelor's degree |
|  | Post-bachelor’s degree |
| **4** | **How old is your child? (Open-ended question)** |
| **5** | **Does your child have a disability?** |
|  | Yes |
|  | No |
| **6** | **Where did your child receive dental care?** |
|  | Private dental clinic |
|  | Public dental clinic (hospital or clinic affiliated with SSR) |
|  | He/She 's never been to the dentist |
| **7** | **How do you think is his/her cooperation during dental care?** |
|  | Totally uncooperative |
|  | Poorly cooperative |
|  | A little cooperative |
|  | Quite cooperative |
|  | Very cooperative |
|  | He/She 's never been to the dentist |
| **8** | **Were you already familiar with advanced behavior management techniques before today?** |
|  | Yes |
|  | No |
| **9** | **How do you think about ACTIVE PROTECTIVE STABILIZATION during ROUTINE dental care?** |
|  | Totally unacceptable |
|  | Unacceptable |
|  | Indifferent |
|  | Acceptable |
|  | Totally acceptable |
| **10** | **How do you think about ACTIVE PROTECTIVE STABILIZATION during EMERGENCY dental care?** |
|  | Totally unacceptable |
|  | Unacceptable |
|  | Indifferent |
|  | Acceptable |
|  | Totally acceptable |
| **11** | **How do you think about PASSIVE PROTECTIVE STABILIZATION during ROUTINE dental care?** |
|  | Totally unacceptable |
|  | Unacceptable |
|  | Indifferent |
|  | Acceptable |
|  | Totally acceptable |
| **12** | **How do you think about PASSIVE PROTECTIVE STABILIZATION during EMERGENCY dental care?** |
|  | Totally unacceptable |
|  | Unacceptable |
|  | Indifferent |
|  | Acceptable |
|  | Totally acceptable |
| **13** | **How do you think about CONSCIOUS SEDATION during ROUTINE dental care?** |
|  | Totally unacceptable |
|  | Unacceptable |
|  | Indifferent |
|  | Acceptable |
|  | Totally acceptable |
| **14** | **How do you think about CONSCIOUS SEDATION during EMERGENCY dental care?** |
|  | Totally unacceptable |
|  | Unacceptable |
|  | Indifferent |
|  | Acceptable |
|  | Totally acceptable |
| **15** | **How do you think about DEEP SEDATION or GENERAL ANESTHESIA during ROUTINE dental care?** |
|  | Totally unacceptable |
|  | Unacceptable |
|  | Indifferent |
|  | Acceptable |
|  | Totally acceptable |
| **16** | **How do you think about DEEP SEDATION or GENERAL ANESTHESIA during EMERGENCY dental care?** |
|  | Totally unacceptable |
|  | Unacceptable |
|  | Indifferent |
|  | Acceptable |
|  | Totally acceptable |
| **17** | **Have you ever allowed advanced behaviour management techniques to be used on your child to carry out dental care?** |
|  | Yes |
|  | No |
| **18** | **If yes, which one?** |
|  | Active protective stabilization |
|  | Passive protective stabilization |
|  | Conscious sedation |
|  | Deep sedation or general anaesthesia |
| **19** | **Now that you know the different advanced behaviour management techniques, would you have preferred to use another one?** |
|  | Yes |
|  | No |
|  | **Total** |

File S3: Variables re-coding in numerical variables

|  | **Question** | **Code** |
| --- | --- | --- |
| **1** | **Who is filling this questionnaire?** |  |
|  | Mather | 0 |
|  | Father | 1 |
| **2** | **Which region are you from?** |  |
|  | Europe |  |
|  | Asia |  |
|  | North America |  |
|  | South America |  |
|  | Africa |  |
|  | Oceania |  |
| **3** | **What is your level of education?** |  |
|  | Primary school degree |  |
|  | Middle school degree |  |
|  | High school degree |  |
|  | Bachelor's degree |  |
|  | Post-bachelor’s degree |  |
| **4** | **How old is your child?** | Open-ended question |
| **5** | **Does your child have a disability?** |  |
|  | Yes | 1 |
|  | No | 0 |
| **6** | **Your child is being treated at...** |  |
|  | Private dental clinic |  |
|  | Public dental clinic (hospital or clinic affiliated with SSR) |  |
|  | He/She never went to the dentist |  |
| **7** | **How do you think is his/her cooperation during dental care?** |  |
|  | Not at all cooperative | 1 |
|  | Poorly cooperative | 2 |
|  | A little cooperative | 3 |
|  | Quite cooperative | 4 |
|  | Very cooperative | 5 |
|  | He/She never went to the dentist | 0 |
| **8** | **Were you already familiar with advanced behavior management techniques before today?** |  |
|  | Yes | 1 |
|  | No | 0 |
| **9** | **How do you think about ACTIVE PROTECTIVE STABILIZATION during ROUTINE dental care?** |  |
|  | Totally unacceptable | 1 |
|  | Unacceptable | 2 |
|  | Indifferent | 3 |
|  | Acceptable | 4 |
|  | Totally acceptable | 5 |
| **10** | **How do you think about ACTIVE PROTECTIVE STABILIZATION during EMERGENCY dental care?** |  |
|  | Totally unacceptable | 1 |
|  | Unacceptable | 2 |
|  | Indifferent | 3 |
|  | Acceptable | 4 |
|  | Totally acceptable | 5 |
| **11** | **How do you think about PASSIVE PROTECTIVE STABILIZATION during ROUTINE dental care?** |  |
|  | Totally unacceptable | 1 |
|  | Unacceptable | 2 |
|  | Indifferent | 3 |
|  | Acceptable | 4 |
|  | Totally acceptable | 5 |
| **12** | **How do you think about PASSIVE PROTECTIVE STABILIZATION during EMERGENCY dental care?** |  |
|  | Totally unacceptable | 1 |
|  | Unacceptable | 2 |
|  | Indifferent | 3 |
|  | Acceptable | 4 |
|  | Totally acceptable | 5 |
| **13** | **How do you think about CONSCIOUS SEDATION during ROUTINE dental care?** |  |
|  | Totally unacceptable | 1 |
|  | Unacceptable | 2 |
|  | Indifferent | 3 |
|  | Acceptable | 4 |
|  | Totally acceptable | 5 |
| **14** | **How do you think about CONSCIOUS SEDATION during EMERGENCY dental care?** |  |
|  | Totally unacceptable | 1 |
|  | Unacceptable | 2 |
|  | Indifferent | 3 |
|  | Acceptable | 4 |
|  | Totally acceptable | 5 |
| **15** | **How do you think about DEEP SEDATION or GENERAL ANESTHESIA during ROUTINE dental care?** |  |
|  | Totally unacceptable | 1 |
|  | Unacceptable | 2 |
|  | Indifferent | 3 |
|  | Acceptable | 4 |
|  | Totally acceptable | 5 |
| **16** | **How do you think about DEEP SEDATION or GENERAL ANESTHESIA during EMERGENCY dental care?** |  |
|  | Totally unacceptable | 1 |
|  | Unacceptable | 2 |
|  | Indifferent | 3 |
|  | Acceptable | 4 |
|  | Totally acceptable | 5 |
| **17** | **Have you ever allowed advanced behaviour management techniques to be used on your child to carry out dental care?** |  |
|  | Yes | 1 |
|  | No | 0 |
| **18** | **If yes, which one?** |  |
|  | Active protective stabilization |  |
|  | Passive protective stabilization |  |
|  | Conscious sedation |  |
|  | Deep sedation or general anaesthesia |  |
| **19** | **Now that you know the different advanced behaviour management techniques, would you have preferred to use another one?** |  |
|  | Yes | 1 |
|  | No | 0 |
